# Entry at a Medical School

**Published:** 1898-09-03

**Authors:** 


### Entry at a Medical School.

The first thing that a lad intended for the medical profession has to do is to pass his preliminary or entrance examination.

The General Medical Council has decided that five years must be spent by the student in the study of his profession before he is allowed to enter for his final examination, and no time of study can be counted as part of these five years until the entrance examination has been passed and the student's name has been placed upon the register.

The subjects of this examination are as follows:—

I. English language, including grammar and composition.

II. Latin, including grammar, translation from specified authors, and translation of easy passages not taken from such authors.

III. Mathematics, comprising (*a*) arithmetic; (*b*) algebra, as far as simple equations, inclusive; (*c*) geometry, the subject-matter of Euclid, Books I, II., and III., with easy deductions.

IV. One of the following optional subjects: (*a*) Greek, (*b*) French, (*c*) German, (*d*) Italian, (*e*) any other modern language, (*f*) logic.

The Council will not accept any certificate of pass in preliminary examination in general education unless the whole of the subjects included in the preliminary examination required by the Council for registration of students of medicine have been passed at the same time.

A University examination required for graduation in arts, wherein the specified subjects of general education are included, may be recognised for the purpose of registration.

The list of examining bodies whose certificates are accepted by the General Medical Council in regard to this examination is a long one, and can be obtained on application to the Registrar, the General Medical Council, 299, Oxford Street, London, W.

Sometimes the Medical Council will accept an examination which is not on the list, so if a student has passed an examination including all the subjects mentioned above, but at some institution not mentioned in the list, he should at once inquire whether the Council will accept this examination as a qualification for registration.

It is not altogether a matter of indifference which of these examinations is taken, because some of the qualifying bodies, especially the Universities, have their own entrance examinations also, which they require to be passed in any case, so that when it is decided what qualification is going to be obtained the selected institution should be written to, to ascertain what entrance examination, recognised by the General Medical Council, will be accepted by it. For example, it is no use going to the trouble of passing the College of Preceptors' entrance examination if one is going in for M.B., because the University would require its own entrance examination to be passed, which itself would be sufficient for registration as a student.

For London students, there can be no doubt at all that matriculation at the London University is the best entrance to the schools, as it is accepted by any of the London qualifying bodies, and leaves the student free to go on to his degree later if he so chooses. The same examination is also accepted in place of the Preliminary Examination in Arts at the University of Durham.

Those London students who do not matriculate usually pass the examination of the College of Preceptors, which is a much simpler affair, but does not admit to the Universities. Full particulars may be obtained from the Secretary, the College of Preceptors, Queen's Square, Bloomsbury, W.C.

It should be pointed out, that since the General Medical Council has made a five years' curriculum compulsory upon all medical students, it takes no longer time to obtain a degree at a University than to obtain the diplomas of the London colleges.

This is of especial importance, in view of the fact that it is quite possible to divide one's time of study between London and another University. At the University of Durham one year's residence is required; at Aberdeen, Edinburgh, and Glasgow, and at the Victoria University two years are required; and at both Oxford and Cambridge about three years. The rest of the five years may in all cases be spent in London. One caution, however, should be given in regard to this—namely, to make sure that the hospital in London at which the medical courses are taken will admit the "foreign" student to all the advantages, such as dresserships, house surgeoncies, &c., which are open to those who have spent all their time there.

If the student so desires he can on registration enter at once upon his studies at a medical school. There are certain subjects, however—namely, chemistry and physics, practical chemistry, practical pharmacy, and elementary biology—which may be studied at various institutions other than medical schools, a certain amount of time occupied in such study being counted as part of the five years' curriculum. The examination in these subjects also may be passed before joining a medical school but not before registration. If, then, it should so happen that a lad is able to pass his entrance examination exceptionally early, and his school happens to be on the list, it is sometimes an advantage not to remove him until he has got these preliminary subjects done with; while, even if one's school is not on the list, expense can sometimes be saved by going to one of these recognised institutions or instructors for these preliminary subjects, life at a school often being cheaper than life at a college.

As a rule, however, as soon as the entrance examination has been passed the student will join his medical school, the dean of which will see after his registration as a medical student. The exact course of study to be followed at the medical school varies somewhat according to the qualification which is desired, and full information on this subject will always be given by the dean of the school; but we would again reiterate the advice so to arrange the course of study and the course of the examinations that whatever other diploma may be taken it shall be possible to obtain a University degree if the occasion for it should arise.

